# Designing and analysing pragmatic clinical trials of complex interventions in mental health: challenges and solutions

**DOI:** 10.1186/1745-6215-12-S1-A149

**Published:** 2011-12-13

**Authors:** Amanda J Farrin, Michelle Collinson

**Affiliations:** 1 Clinical Trials Research Unit, University of Leeds, Leeds, LS2 9JT, UK

## Objectives

Designing and analysing pragmatic clinical trials involving complex interventions is becoming increasingly complex, particularly in the field of mental health. This paper will describe a variety of challenges encountered during the design of a complex intervention mental health trial and the decisions made on how to overcome these challenges. The objectives are to share knowledge gained through the design of this large multi-centre trial and to facilitate discussion and widen the debate regarding potential issues and possible solutions in the design of such trials.

## Methods

The HTA-funded SHIFT Trial (Self-Harm Intervention, Family Therapy) is a pragmatic, randomised, controlled trial, comparing family therapy with treatment as usual in 832 adolescents who have self-harmed at least twice, with a primary outcome of repetition of self-harm leading to hospital attendance. The particular challenges encountered during the trial design and solutions adopted will include the level of randomisation, therapist variation, multiple methods employed to collect the primary outcome, maximising reliable data collection and the planned analytical techniques.

## Results

In the SHIFT trial different therapists are involved in delivering interventions, therefore we will explain the rationale for choosing individual over cluster randomisation and methods adopted to minimise therapist variation. In trials of complex interventions dropout from treatment and follow-up is common, so we will discuss the multiple methods employed to collect the primary outcome and how these maximise reliable data collection. Due to the nature of SHIFT’s endpoints, the analysis will use techniques not often encountered in mental health trials. We will discuss the planned interim analysis; the use of Cox’s Proportional Hazards Model to test for differences in repetition rates and the use of multilevel survival frailty models[1] to determine the extent of clustering on outcome due to therapists and the impact on the precision of the treatment effect. Multiple events analysis using Anderson-Gill[2] methodology making use of the timing and number of first and subsequent events will be discussed.

## Conclusions

We will show how it is possible to design solutions to the challenges encountered in the design of complex trials in mental health, that are feasible, practical and robust.
